# Cysteine import contributes to hypochlorous acid resistance in *Staphylococcus aureus*

**DOI:** 10.1128/msphere.00214-26

**Published:** 2026-08-24

**Authors:** Abigail G. Hall, Aliyah J. Collins, Anna Sommers, Ryan Singh, Sasmita Panda, Sujata S. Chaudhari, Andrew J. Monteith, Vinai C. Thomas

**Affiliations:** 1 Department of Pathology, Microbiology and Immunology, Center for Staphylococcal Research, University of Nebraska Medical Center 198515, Omaha, Nebraska, USA; 2 Department of Microbiology, University of Tennessee 189504, Knoxville, Tennessee, USA; The University of Iowa, Iowa City, Iowa, USA

**Keywords:** *Staphylococcus aureus*, L-cysteine, L-cystine, bacillithiol, hypochlorite, stress

## Abstract

Hypochlorous acid (HOCl) is a potent antimicrobial oxidant produced by innate immune cells to eliminate invading bacterial pathogens, including *Staphylococcus aureus*. HOCl readily oxidizes thiols, amines, and macromolecules in *S. aureus*. Yet, some bacterial cells within an HOCl-challenged population withstand this oxidative stress and proceed to establish infection. The mechanisms that enable *S. aureus* to resist the effects of HOCl remain incompletely understood. Here, we show that cysteine uptake is a major determinant of HOCl resistance in *S. aureus*. Inactivation of the cysteine transporters *tcyABC* and *tcyP* resulted in a dose-dependent increase in sensitivity to HOCl. Cysteine uptake through these transporters helped maintain bacillithiol homeostasis and limit oxidative damage to protein thiols during HOCl stress. Importantly, cysteine uptake was required for the survival of *S. aureus* during interaction with activated human neutrophils that generate HOCl to kill phagocytosed bacteria. These findings reveal that cysteine uptake expands the thiol buffering capacity of *S. aureus* and functions as a major mechanism for scavenging HOCl, which may contribute to its persistence in the infected host.

**IMPORTANCE:**

*Staphylococcus aureus* is a major human pathogen that causes recurrent and antibiotic-recalcitrant infections. Understanding the mechanisms that allow *S. aureus* to evade key innate immune defenses is therefore of considerable interest for developing effective intervention strategies. Our findings show that cysteine uptake is a critical component of *S. aureus* defense against hypochlorous acid (HOCl) produced by innate immune cells. Cysteine functions as a reactive sink for HOCl and thereby prevents depletion of both low-molecular-weight thiols and protein thiols in *S. aureus*.

## OBSERVATION

*Staphylococcus aureus* is a versatile pathogen capable of causing a broad range of diseases from skin and soft tissue infections to more life-threatening illnesses such as endocarditis and sepsis (1). To establish an infection, *S. aureus* must overcome the early innate immune response of the host. Shortly after invasion, tissue-resident macrophages recognize *S. aureus* and initiate an inflammatory cascade that rapidly recruits neutrophils, the key effector cells responsible for early bacterial clearance (2). Activated neutrophils engulf *S. aureus* into phagosomes where they get exposed to hypochlorous acid (HOCl), a highly potent bactericidal oxidant generated by myeloperoxidase (MPO) activity (3, 4). Kinetic modeling studies predict that MPO generates HOCl within the mature phagosome at rates exceeding 100 mM min^−1^. However, because HOCl is highly reactive, it is not expected to accumulate as a free oxidant. Instead, the majority of HOCl reacts almost instantaneously with phagosomal proteins, while only a smaller fraction reaches bacterial targets near the site of MPO activity (4, 5). Despite this potent oxidative host defense, *S. aureus* survives neutrophil attack and frequently establishes infection. The mechanisms that contribute to HOCl resistance in *S. aureus* remain poorly understood.

Although HOCl can modify many types of biomolecules, its primary targets are thiols and amines within proteins and free amino acids (6). Both α-amines and side-chain amines of amino acids within proteins are susceptible to chlorination and form chloramines (6). Similarly, thiols are initially chlorinated by HOCl and subsequently converted to sulfenic acids upon reaction with water (6). The sulfenic acid can react with a nearby cysteine within the same protein or with other low-molecular-weight (LMW) thiols such as free L-cysteine (L-Cys) or bacillithiol (BSH), resulting in S-cysteinylation or S-bacillithiolation, respectively (7).

**Fig 1:**
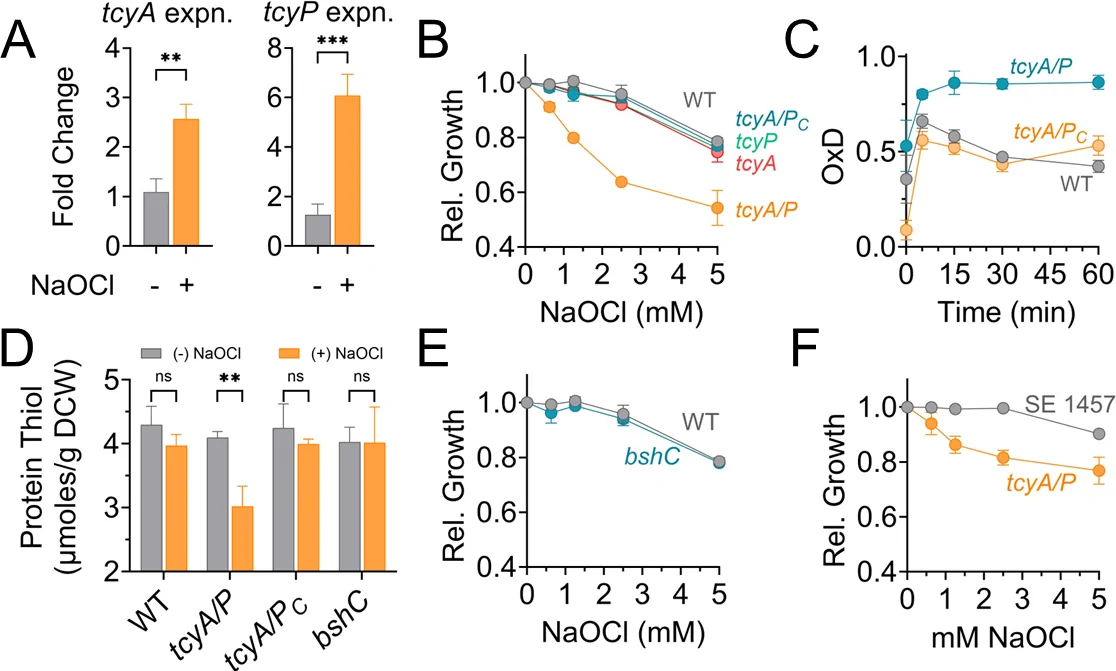
L-Cys influx counters hypochlorite stress. (**A**) RT-qPCR analysis of *tcyA* and *tcyP* expression following 10 min treatment of *S. aureus* with 2 mM NaOCl in tryptic soy broth (TSB), pH 7.2. Fold change in gene expression was calculated relative to the untreated control. *n* = 3, mean ± SD, Student’s *t*-test. (**B**) Dose-dependent impact of sodium hypochlorite (NaOCl) on growth of *S. aureus* in TSB media. The relative growth (*y*-axis) represents the ratio of the area under the growth curve for cultures treated with NaOCl to that of the untreated control, (−) NaOCl. (**C**) Degree of oxidation (OxD) of bacillithiol as a function of time following 2 mM NaOCl treatment in TSB. NaOCl was treated at time 0. (**D**) The protein thiol content in *S. aureus* was determined using the DTNB assay. g DCW, gram dry cell weight, *n* = 3, mean ± SD, two-way ANOVA, Tukey’s post-test. (**E and F**) Relative growth of various strains following NaOCl challenge. SE1457, *S. epidermidis* 1457. *tcyA*/*P*_C_, *tcyA*/*P* complemented with *tcyABC* through integration at the SaPI site. **P ≤ 0.01, ***P ≤ 0.001.

Given the strong reactivity of HOCl with thiols, we hypothesized that uptake of L-cystine (L-CySS) by *S. aureus* and its eventual reduction to L-Cys in the cytosol may act as a critical defense mechanism against HOCl stress. Indeed, a previous study showed that both genes encoding L-CySS/L-Cys transporters (*tcyABC* and *tcyP*) are upregulated following HOCl stress (8, 9), a finding that we independently confirmed (Fig. 1A). To test whether these transporters contribute to HOCl resistance, we assessed the sensitivity of wild-type (WT), *tcyA*, *tcyP*, and the *tcyAtcyP* double mutant (hereafter referred to as *tcyA*/*P*) to HOCl generated from sodium hypochlorite, pH 7.2 (Fig. 1B). The WT strain displayed a dose-dependent decrease in growth as the hypochlorite concentration increased (Fig. 1B). Although the *tcyA* and *tcyP* single mutants behaved similarly to the WT strain, the *tcyA*/*P* double mutant showed a more prominent growth defect against hypochlorite (Fig. 1B). Furthermore, the impaired growth of the *tcyA*/*P* double mutant was restored upon complementation with *tcyABC* (see *tcyA*/*P*_C_ strain), indicating that the phenotype did not arise from off-target effects (Fig. 1B). These findings suggest that the uptake and accumulation of L-Cys in the *S. aureus* cytosol contribute to HOCl resistance and that the two L-CySS/L-Cys transporters compensate for each other under HOCl stress.

At a mechanistic level, the importance of L-Cys for HOCl resistance is highlighted by two key findings. First, L-CySS uptake was critical for maintaining the redox potential of BSH following hypochlorite stress (Fig. 1C). We monitored the OxD and estimated the corresponding redox potential of BSH (*E*_BSH_) from WT, *tcyA*/*P* mutant, and *tcyA*/*P*_C_ strains harboring the Brx-roGFP2 biosensor following 2 mM sodium hypochlorite challenge. In the WT strain, HOCl rapidly oxidized BSH, shifting *E*_BSH_ from −288 mV to −271 mV within 5 min, after which it gradually returned to a reduced state and approached baseline (−284 mV) by 1 h (Fig. 1C). In contrast, inactivation of the L-CySS/L-Cys transporters not only increased basal BSH oxidation (*E*_BSH_ shifted from −288 mV to −278 mV) but, more importantly, also prevented recovery of *E*_BSH_ over time under HOCl stress (Fig. 1C). Second, free L-Cys played an important role in limiting protein thiol oxidation by HOCl (Fig. 1D). The intracellular protein thiol content from bacterial cell pellets after removal of LMW thiols was quantified using Ellman’s reagent, or DTNB (5,5′-dithiobis-(2-nitrobenzoic acid)) (10). We found that the *tcyA*/*P* double mutant contained significantly fewer protein thiols than the WT strain, indicating increased protein thiol oxidation in the absence of L-CySS import (Fig. 1D). Both the oxidation of BSH and protein thiols in the *tcyA*/*P* mutant could be readily complemented (Fig. 1C and D). The rapidity by which L-Cys influx blunts HOCl-mediated oxidation of cellular thiols strongly indicates a role for L-Cys as an important sink for HOCl.

An alternative explanation on the need for increased L-CySS uptake during HOCl stress may result from the use of L-Cys as a precursor for BSH biosynthesis (11). BSH was previously shown to be oxidized by HOCl (12). To test this possibility, we determined the sensitivity of the *bshC* mutant to HOCl stress. The *bshC* gene encodes a cysteine ligase that catalyzes the addition of L-Cys to GlcN-Mal (11), the final step of BSH biosynthesis. Surprisingly, under HOCl stress, growth of the *bshC* mutant was similar to the WT strain (Fig. 1E). Also, the *bshC* mutant did not exhibit increased protein thiol oxidation compared to WT (Fig. 1D). These findings strongly suggest that L-Cys plays a more direct and prominent role as an LMW thiol in defending against HOCl stress than BSH. Furthermore, the significance of these findings is underscored by the observation that L-Cys-dependent HOCl resistance is not unique to *S. aureus* but is also conserved in other staphylococcal species such as *S. epidermidis* (Fig. 1F).

**Fig 2:**
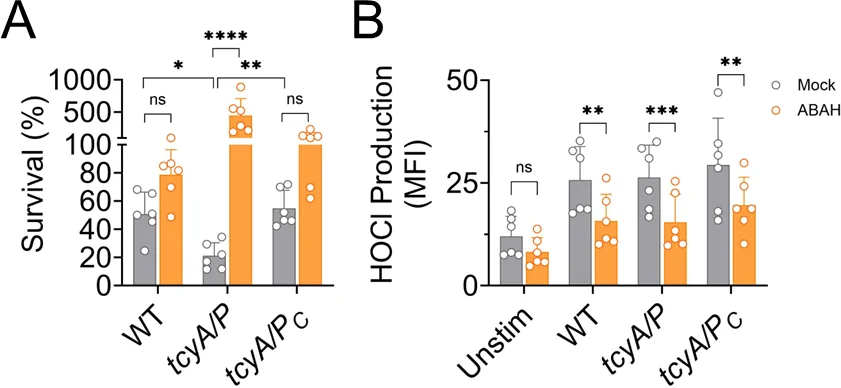
L-CySS/L-Cys transporters of *S. aureus* are essential for surviving human neutrophil-mediated killing. (**A**) Opsonized *S. aureus* was incubated with neutrophils (±250 µM ABAH) in DMEM supplemented with 10% heat-inactivated FBS at an MOI of 1 for 4 h, and bacterial viability was determined by spot plating on TSA. Percent *S. aureus* survival in the presence of neutrophils is quantified relative to the strain cultured in the absence of neutrophils (100% = no neutrophil killing, <100% = neutrophil killing, and >100% = growth enhancement in the presence of neutrophils). Survival data were log transformed prior to statistical analysis to account for the increasing variance observed at higher survival values. *n* = 6, mean ± SD, one-way ANOVA, Sidak’s post-test. (**B**) Neutrophils were stained with BioTracker 574 Red HOCl, approximately 15 min prior to fixation in 4% PFA. Neutrophils were blocked and stained with anti-CD15 and anti-CD16 antibodies, and the mean fluorescence intensity (MFI) for BioTracker 574 Red HOCl in the neutrophil population was quantified by flow cytometry. MFI was normalized to unstained neutrophils. *n* = 6, mean ± SEM, one-way ANOVA, Sidak’s post-test, *P ≤ 0.05, **P ≤ 0.01, ***P ≤ 0.001, ****P ≤ 0.0001.

Finally, we determined whether L-Cys import in *S. aureus* contributes to resistance against human neutrophils that mediate killing via HOCl stress (Fig. 2A). We incubated opsonized *S. aureus* with human neutrophils and measured bacterial survival. Under these conditions, both the WT and *tcyA*/*P* mutant stimulated a robust and equivalent HOCl response in human neutrophils (Fig. 2B). However, the *tcyA*/*P* mutant exhibited much reduced survival in the presence of neutrophils than the WT strain, a phenotype that was readily complemented (Fig. 2A). More importantly, treatment with 4-aminobenzoic acid hydrazide (ABAH), a selective inhibitor of MPO-mediated HOCl production, rescued the survival defect of the *tcyA/P* mutant, indicating that the phenotype is primarily driven by HOCl. These findings suggest that *S. aureus* actively utilizes L-CySS/L-Cys import as a mechanism to counter HOCl-mediated bactericidal activity of neutrophils.

In *S. aureus*, MerA, a flavin disulfide reductase, was proposed to directly reduce HOCl and decrease its lethality (7). The expression of *merA* is under the control of HypR, an Rrf2-family transcriptional repressor that is responsive to HOCl stress (8). In addition, BSH has also been implicated in HOCl resistance (12). Although BSH is the most abundant LMW thiol in *S. aureus*, the disproportionately high concentration of HOCl required to oxidize BSH suggests that other cellular components are targeted before BSH (13). Thus, it was concluded that BSH was not likely to be a sink for HOCl but rather helps through S-bacillithiolation of proteins (13). In this study, we suggest that L-Cys derived from L-CySS import serves as an important and direct scavenger of HOCl.

How does an influx of L-Cys enable *S. aureus* to defend against HOCl stress? LMW thiols act as a redox buffer due to their high concentration within cells and rapid reaction with oxidants. In *S. aureus*, the intracellular concentration of BSH is approximately 1.2 mM during exponential growth in TSB (assuming 1.55 mL/g dry weight of intracellular water) (12, 14). By comparison, intracellular L-Cys levels are ~10 times lower, estimated at 0.13 mM (12). Despite the marked difference in intracellular concentration of these LMW thiols, BSH is predicted to react with HOCl only about twofold more effectively than L-Cys, since the rate constant of the HOCl reaction with L-Cys (*k*_Cys_, 3.6 × 10^8^ M^−1^ s^−1^) is higher than that of BSH (*k*_BSH_, 6 × 10^7^ M^−1^ s^−1^) (13, 15).

The critical dependence of *S. aureus* on L-CySS/L-Cys transporters for survival under HOCl stress suggests that HOCl triggers a substantial increase in L-Cys influx. We speculate that intracellular L-Cys concentrations during HOCl stress will significantly rise to outcompete the reaction of BSH with HOCl, effectively emerging as a major thiol sink for HOCl. Thus, inducible L-CySS uptake may provide *S. aureus* with a rapid and adaptable mechanism to expand its thiol buffering capacity and mitigate HOCl-mediated oxidative damage. Altogether, we contend that L-Cys influx helps *S. aureus* resist neutrophil-derived HOCl and supports its ability to cause infection.

## SUPPLEMENTAL MATERIAL

Supplemental Material msphere.00214-26-s0001.docx Supplemental materials and methods and Table S1.

ASM does not own the copyrights to Supplemental Material that may be linked to, or accessed through, an article. The authors have granted ASM a non-exclusive, world-wide license to publish the Supplemental Material files. Please contact the corresponding author directly for reuse.
