# Supplementary material for: Cysteine import contributes to hypochlorous acid resistance in Staphylococcus aureus
Source: mSphere. 2026 Aug 24;11(9):e00214-26. doi: 10.1128/msphere.00214-26 (PMC13621853; doi:10.1128/msphere.00214-26)
Supplement: Supplementary file 1 [file msphere.00214-26-s0001.docx]

**Supplemental Materials and Methods**

**Bacterial strains, plasmids and growth conditions**

The bacterial strains and plasmids used in this study are listed in Table S1. *S. aureus* strains were grown in tryptic soy broth (TSB; Difco Laboratories). Bacterial growth was carried out at 37°C, starting from an initial OD₆₀₀ of 0.06, either in flasks or in 96-well microtiter plates. When cultures were grown in flasks, a 10:1 flask-to-volume ratio and agitation at 250 rpm was maintained to ensure adequate aeration. Microtiter plate cultures were incubated in a Tecan Infinite M200 spectrophotometer at 37°C and maximum agitation. *Escherichia coli* ElectroTen Blue was used for routine plasmid maintenance and propagation, and cultures were incubated aerobically in Luria–Bertani (LB) media. Antibiotics were added as necessary at the following concentrations: Ampicillin, 100 µg/mL; Erythromycin, 5 µg/mL.

HOCl stress was induced by adding sodium hypochlorite (NaOCl) directly to TSB, with the pH adjusted to 7.2. For bacterial growth assays involving HOCl challenge, cultures were grown in TSB containing two-fold serial dilutions of NaOCl (0, 0.625–5 mM) to assess dose-dependent effects on growth. Growth data were analyzed as relative growth, defined here as the ratio of the area under the growth curve (AUC) for the test condition ((+) NaOCl) to that of the untreated control ((-) NaOCl). Relative growth values were then plotted as a function of NaOCl concentration.

**DNA manipulation and strain construction**

*S. aureus* JE2 and its isogenic mutants were obtained from the Nebraska Transposon Mutant Library (NTML). All transposon insertions were re-transduced into the parental JE2 background using ϕ11 bacteriophage to eliminate potential suppressor mutations. The double mutants were generated by first replacing the Ermᴿ cassette of the transposon insertion with a Kanᴿ cassette, followed by transduction of the modified transposon into the desired NTML strain. The complementation of mutant strains was achieved by integrating the full-length gene(s), under the control of its native promoter, into the SaPI1 site within the *S. aureus* chromosome using pJC1111 integration vector (1).

**Degree of oxidation (OxD) of BSH**

The OxD was determined as previously reported (2, 3). Briefly, *S. aureus* strains expressing the Brx-roGFP2 biosensor were grown aerobically to an OD_600_ of 1.0. Fluorescence measurements were taken at 0, 5, 15, 30 and 60 minutes after the addition of 2mM NaOCl. Fully reduced and oxidized controls were prepared with 10 mM DTT and 5 mM diamide, respectively. Brx-roGFP2 biosensor fluorescence emission was measured at 510 nm after excitation at 395 and 475 nm using the Tecan Infinite M200 spectrophotometer. The OxD of the Brx-roGFP2 biosensor was determined for each sample and normalized to fully reduced and oxidized controls (eq. 1). Based on the OxD and 𝐸^0′^𝑟𝑜𝐺𝐹𝑃2 = -280 mV, the BSH redox potential (E_BSH_) was calculated according to the Nernst equation (eq. 2). Any samples which had a calculated OxD >1, the values were capped at 0.99.

$$(I₃₉₅₍sample₎ \times I₄₇₅₍red₎ - I₃₉₅₍red₎ \times I₄₇₅₍sample₎)$$

$OxD = ─────────────────────────────────────────────$	*eq. 1*

$$I₃₉₅₍sample₎ \times I₄₇₅₍red₎ - I₃₉₅₍sample₎ \times I₄₇₅₍ox₎ + I₃₉₅₍ox₎ \times I₄₇₅₍sample₎ - I₃₉₅₍red₎ \times I₄₇₅₍sample₎$$

where ‘I’ represents the observed fluorescence excitation intensities.

$E_{\left\{ BSH \right\}}= E_{\left\{ roGFP2 \right\}}= E_{\left\{ roGFP2 \right\}}^{0'}- \left( \frac{RT}{\left( 2F \right)} \right)*\ln\left( \frac{\left( 1 - OxD \right)}{OxD} \right)$	*eq. 2*

**DTNB Assay**

Protein thiols were measured as described previously with minor changes (3). *S. aureus* strains were grown to a OD_600_ of 1.0 and treated with 2 mM NaOCl for 30 minutes. 25 OD_600_ units of NaOCl-treated culture sample were collected and brought to a final volume of 30 mL using PBS and centrifuged for 5 minutes at 10,000 × g and the pellet was resuspended in 600 μL sample buffer containing 50% acetonitrile in 20 mM Tris-HCl, pH 8.0 (v/v). The suspension was incubated for 10 min at 60 °C, and centrifuged for 10 minutes at 10,000 × g. The remaining pellet was resuspended in 1 mL denaturing buffer, consisting of 8 M urea, 1% CHAPS (3-((3-cholamidopropyl)-dimethylammonio)-1-propanesulfonate), 1 mM EDTA, 200 mM Tris-HCl (pH 8.0), and 10 μL of 100 mM DTNB was added directly. The suspension was incubated for 15 min at 60 °C and centrifuged 5 minutes at 10,000 × g. The supernatant was diluted 1:2, A_412_ was measured, and the protein thiol content was calculated using the molar absorption coefficient, 14,150 M^-1^ cm^-1^.

**Quantitative Reverse-Transcriptase PCR (RT-qPCR) analysis**

RT-qPCR was performed using the ΔΔCT method with housekeeping *sigA* as internal control, as described previously (4).

**Neutrophil killing assay**

Human neutrophils were isolated from healthy donors. Written informed consent was obtained from all donors, and experimentation performed on these samples was approved by the University of Tennessee Knoxville Institutional Review Board (IRB-22-07231-XP). The neutrophils were seeded in 96-well plates at 2.5 × 10^5^ cells/well in DMEM supplemented with 10% heat-inactivated FBS and rested at 37°C with 5% CO₂ for 45 minutes. Where indicated, 4-aminobenzoic hydrazide (ABAH) was added to neutrophils at a final concentration of 250 µM, and cells were incubated for an additional 15 minutes. *S. aureus* strains were opsonized in non-heat-inactivated FBS diluted 1:1 with DMEM before addition to neutrophils at an MOI of 1. Cultures were incubated for 4 hours at 37°C with 5% CO₂, serially diluted, and spot plated onto TSA. Colony-forming units (CFU) were enumerated the following day. Percent *S. aureus* survival was calculated by dividing the average CFU recovered from neutrophil-containing wells by the average CFU recovered from bacteria-only control wells.

**HOCl Flow Cytometry Assay**

Human neutrophils isolated from healthy donors were seeded in 96-well plates at 1.5 × 105 cells/well in DMEM supplemented with 10% FBS and rested at 37°C with 5% CO₂ for 45 minutes. Where indicated, ABAH was added to neutrophils at a final concentration of 250 µM 15 minutes prior to stimulation with *S. aureus* strains at an MOI of 10. Neutrophils were stimulated for 2 hours at 37°C with 5% CO₂. Fifteen minutes prior to fixation, cells were stained with BioTracker 574 Red HOCl dye. Cells were then fixed in 4% PFA, blocked, and stained with anti-CD15 and anti-CD16 antibodies. BioTracker 574 Red HOCl mean fluorescence intensity (MFI) was quantified within the neutrophil population by flow cytometry and normalized to unstained control neutrophils.

**Supplemental Table**

**Table S1: Strains and Plasmids**

| Strain | Description | Source |
| --- | --- | --- |
| *E. coli* Electro10 Blue | *E. coli* strain used for plasmid maintenance | Agilent Tech. |
| *S. aureus* JE2 | *S. aureus* USA300 LAC 13C cured of endogenous plasmids | (5) |
| RN4220 | Restriction deficient strain used as a transformation intermediate | (6) |
| *tcyP* | *Bursa aurealis* transposon mutant, Erm^R^ | (5) |
| *tcyA* | *Bursa aurealis* transposon mutant, Kan^R^ | (3) |
| *tcyA*/*P* | *tcyA*::Tn Kan^R^, *tcyP*::Tn Erm^R^ | (3) |
| *tcyA*/*P* :: *tcyABC* | *tcyA*/*P* mutant complemented with *tcyABC* in SaPI site | (3) |
| *S. epidermidis* 1457 | Clinical isolate (ica+ agr type II) | (4) |
| 1457 *tcyA/P* | Inframe isogenic deletion mutant of 1457 cysteine transporter *tcyA* and *tcyP* | (4) |
| Plasmid |  |  |
| pJC1111 | SaPI integration vector | (1) |
| pVT4 | Brx-roGFP2 under control of constitutive P_1_*sarA* | (3) |
| pAH6.5 | pJC1111:: *tcyABC* | (3) |

**References**

1.	Chen J, Yoong P, Ram G, Torres VJ, Novick RP. 2014. Single-copy vectors for integration at the SaPI1 attachment site for *Staphylococcus aureus*. Plasmid 76:1–7.

2.	Loi VV, Harms M, Muller M, Huyen NTT, Hamilton CJ, Hochgrafe F, Pane-Farre J, Antelmann H. 2017. Real-time imaging of the bacillithiol redox potential in the human pathogen *Staphylococcus aureus* using a genetically encoded bacilliredoxin-fused redox biosensor. Antioxid Redox Signal 26:835–848.

3.	Hall AG, Alqarzaee AA, Collins AJ, Panda S, Romanova S, Chaudhari SS, Monteith AJ, Frees D, Thomas VC. 2026. Activation of the Spx redox sensor counters cysteine-driven Fe(II) depletion under disulfide stress doi:10.7554/elife.110004.1. eLife Sciences Publications, Ltd.

4.	Singh RM, Chaudhari SS, Panda S, Hutfless EH, Heim CE, Shinde D, Alqarzaee AA, Sladek M, Kumar V, Zimmerman MC, Fey PD, Kielian T, Thomas VC. 2023. A critical role for staphylococcal nitric oxide synthase in controlling flavohemoglobin toxicity. Redox Biol 67:102935.

5.	Fey PD, Endres JL, Yajjala VK, Widhelm TJ, Boissy RJ, Bose JL, Bayles KW. 2013. A genetic resource for rapid and comprehensive phenotype screening of nonessential *Staphylococcus aureus* genes. mBio 4:e00537–12.

6.	Kreiswirth BN, Lofdahl S, Betley MJ, O'Reilly M, Schlievert PM, Bergdoll MS, Novick RP. 1983. The toxic shock syndrome exotoxin structural gene is not detectably transmitted by a prophage. Nature 305:709–12.
